# Supplementary material for: Engineering the vibrational coherence of vision into a synthetic molecular device
Source: Nat Commun. 2018 Jan 22;9:313. doi: 10.1038/s41467-017-02668-w (PMC5778125; doi:10.1038/s41467-017-02668-w)
Supplement: Supplementary file 1 — Supplementary information [file 41467_2017_2668_MOESM1_ESM.pdf]

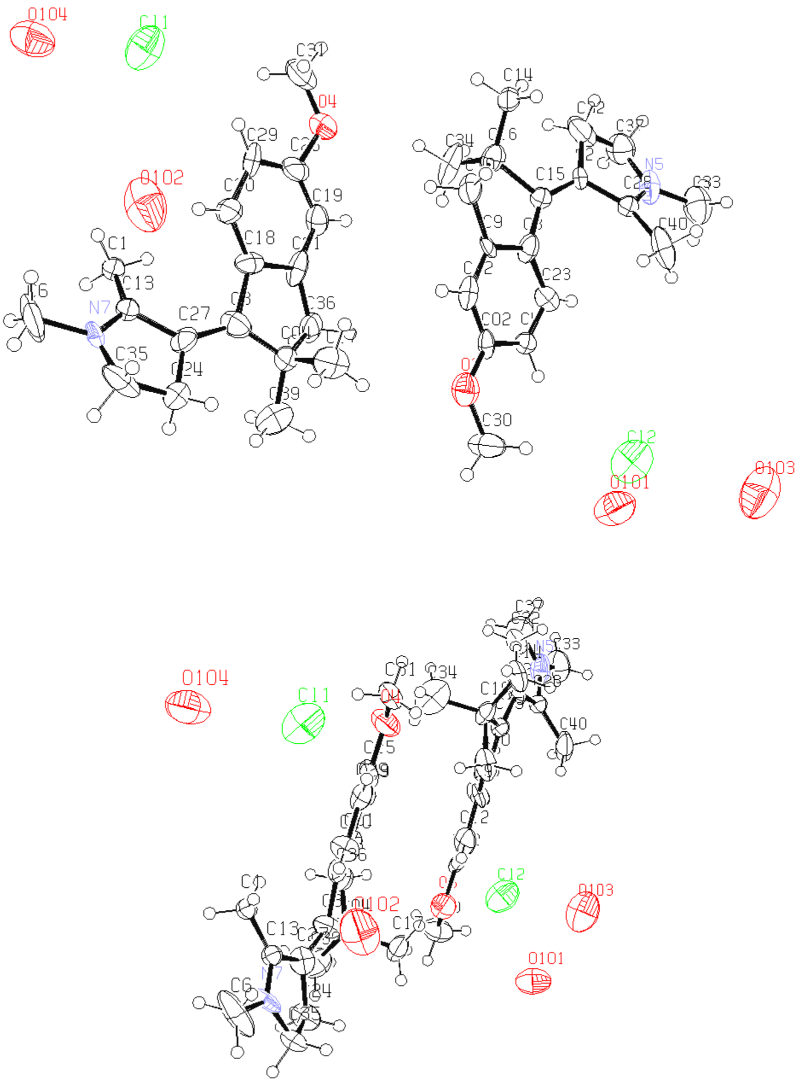

Supplementary Figure 1: Crystallographic structure of compound *Z*-1. Ellipsoids enclose 50% probability. Front perspective view of the molecule (top) and view in the plane of the indanylidene moiety (bottom). Single yellow crystals of compound *Z*-**1** were obtained from a dichloromethane-methanol solution (9: 1 v/v) upon slow evaporation of the solvents in the dark. Single crystal of *Z*-**1** was submitted to X-ray data collection via an Oxford-Diffraction Xcalibur Sapphire 3 diffractometer by using a graphite monochromated Mo-Kα radiation (λ = 0.71073 Å) at 293 K in orthorhombic space group. The structure was solved by direct methods, implemented on SHELXS-2014 program. The refinement was carried out by full-matrix anisotropic least squares on F^2 for all reflections for non-H atoms by means of the SHELXL-2014 program (R1(orb data)=0.11). Unfortunately, the data quality is not suitable for a crystallographic deposit, as several atoms are affected by thermal disorder. Presently, it has not been possible to address this issue. However, the acquired data confirm the chemical structure of *Z*-**1** with the distortion of the pyrroline ring placed outside the plane of the molecule, in line with the computational data (compare with Figure 4a). A comparison between the most relevant computed and observed dihedral angles is given in Supplementary Figure 2a.

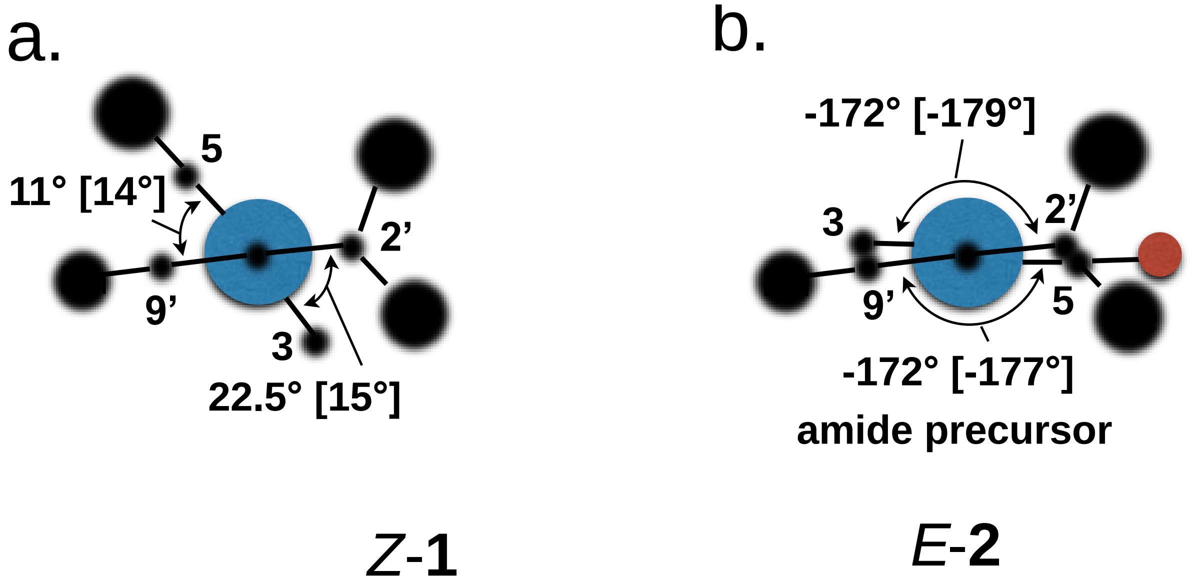

Supplementary Figure 2: Comparison between computed and observed torsional parameters. **a** Values of the C9'-C1'=C4-C5 and C2'-C1'=C4-C3 torsional parameters from the computed (in brackets, see Supplementary Notes 1 and 2 and Supplementary Figures 3 to 5) and available structural data (Supplementary Figure 1) for *Z*-**1**. **b** The same data for *E*-**2**. Notice that, in this case however, the computed parameters (in brackets) are compared with a previously reported [1] precursor of *E*-**2** which is "sterically close" to *E*-**2** (i.e. a H atom is replaced with an O atom)**,** since *E*-**2** could not be crystallized.

Supplementary Note 1: Investigation of the room temperature thermal equilibrium in methanol

In order to theoretically describe the spectroscopic and dynamic properties of the studied molecular switches a QM/MM-based protocol oriented to generate an *average configuration* of the *solvent environment* at the *desired temperature* was used to optimize the ground state geometries and generate initial conditions to initiate exited states dynamics. The proposed protocol [2] is based on the idea of Herbert *et al*. [3,4] of combining the Average Solvent Electrostatic Configuration (ASEC) model [5,6] and the free energy gradient method proposed by M. Nagaoka *et. al.* to optimize solvated molecular systems.[7-9] In this QM/MM model the solute is considered quantum mechanically (QM) while a molecular mechanic force field is used to describe the solvent environment (MM).

As stated by M. Nagaoka *et. al.*, [7-9] the free energy gradient, or the forces *F(q)* acting on the QM part of the system, can be computed as:

$$F(q) = -\frac{\partial G(q)}{\partial q} = -\langle\frac{\partial V}{\partial q}\rangle \approx -\frac{\partial \langle V\rangle}{\partial q},$$

where *q* represents the coordinates of the QM part and *G* is the free energy of the system, which can be computed, in a very good approximation, as the average of the potential interaction, $\langle V\rangle$, between the QM and MM subsystems. In this context, the ASEC model [6,7] is used to compute, in a very efficient way, the average potential interaction $\langle V\rangle$. ASEC model is a mathematical construct called “ASEC configuration” created from a selected sampling of configurations, obtained via extensive molecular dynamics (MD), to mimic the effect of thermodynamic equilibrium conditions at a selected temperature. The solute is kept fixed along the MD in order to sample the environment, being subsequently optimized in the presence of the average external configuration (Supplementary Figure 3a). The GROMACS code [10] was used for the MD, performing an initial thermalization of 1 ns, followed by 4 ns of production in the *NPT* ensemble and standard room conditions. The QM/MM geometry optimization is performed using the quasi-Newton-Raphson method implemented in the MOLCAS-TINKER interface.[11, 12] The QM subsystem is described using Complete Active Space Self Consistent Field (CASSCF) method.[13] For the MM subsystem, OPLS force field parameters are used.[14] Finally, the absorption energies of optimized structures are re-computed using second order perturbation theory (CASPT2)[15] with ionization potential electronic affinity (IPEA) set to zero. An active space comprising of 12 electrons in 11 orbitals is used for all QM calculations in current study.

Since the configurations of the environment selected to generate the ASEC configuration (100 solute-solvent uncorrelated configurations in our case) are obtained from the system into thermodynamic equilibrium conditions, a Boltzmann distribution of the absorption energies is expected to be observed from those structure. Therefore, these 100 configurations can be used to initiate excited state dynamics. For the two molecular switches, absorption bands were constructed (Supplementary Figure 4). A single configuration from each band, representing the average was selected to map the potential energy surfaces. The selected two configurations were re-optimized on the ground state. The setup used for optimization and subsequent QM/MM calculations is shown in Supplementary Figure 3b. The vertical excitation energies and oscillator strengths were computed for the ground state optimized structures. According to oscillator strengths, the transition from ground state to second excited state (S_0 to S_2) was highly probable in both switches. Therefore, minimum energy path (MEP) calculation and Franck-Condon trajectory for each of the two switches were initiated from S_2. The MEP calculation resulted in minima on S_2 and S_1 for *Z*-**1** and *E*-**2** switches respectively (Supplementary Figures 5c and 5d). Starting from these minima, torsional scans for each switch was performed by constraining the dihedrals C9’-C1’=C4-C5 and C2’-C1’=C4-C3 (see Supplementary Figure 6). The Franck-Condon trajectories were computed at 3-root-state-average CASSCF/6-31G*/OPLS level. This was performed using the Dynamix module implemented in the MOLCAS program and more details can be found in Schapiro et al.[16] Since the CASSCF method does not account for dynamic electron correlation, the energy profiles had to be recomputed using a method that doesn’t suffer from this problem. Due to this reason, for each geometry along the trajectories, the energies were recomputed at 4-root-state-average CASPT2 and XMCQDPT2 levels (see Supplementary Note 3 for more details on the latter).

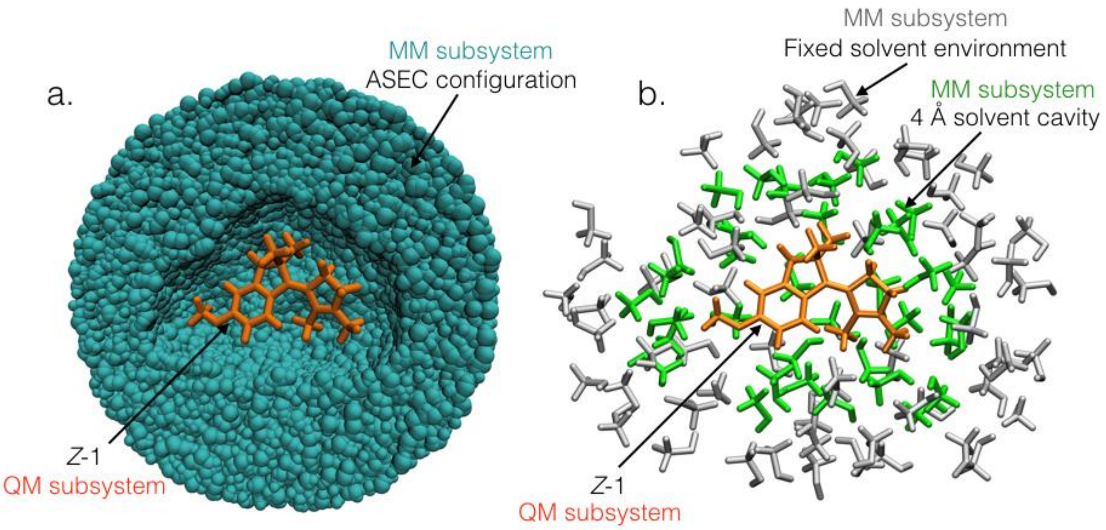

Supplementary Figure 3: QM/MM setups used in current study. a. ASEC cavity hosting *Z*-**1** molecular switch. The switch (orange) is treated at QM level whereas the configurations (green) at MM level. **b.** QM/MM setup used to study the single configurations. The switch (orange) is treated at QM level. Solvent molecules within 4 Å distant are treated as MM active (light green) while the rest (gray) is kept frozen MM level.

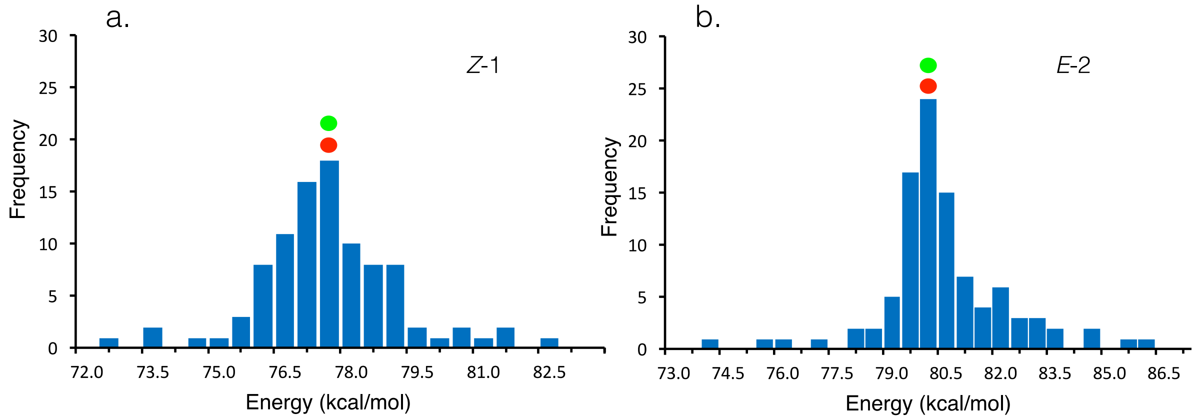

Supplementary Figure 4: Distribution of energies of 100 configurations of *Z*-1 (a.) and *E*-2 (b.) switches. Red and green circles denote energies computed by ASEC and single S_0 configurations representing the ASEC result (see Supplementary Note 2 below) respectively. Notice that the simulated distributions consistently replicate the absorption bands displayed in Figure 1 both in λ_{max} and width.

Supplementary Note 2: Computation of a representative QM/MM structure in solution

Parts a. and b. of Supplementary Figure 5 illustrate the steric clash/pretwist in *Z*-**1** and geared/planar conformation in *E*-**2**. In *Z*-**1**, the steric clash of the nearly eclipsed hydrogens at C3 with the methyl groups at C2′ as well as the clash of the methyl group and hydrogen at C5 and C8′ respectively result in a strongly twisted S_0 equilibrium conformation. Instead structure *E*-**2** displays a substantially planarized S_0 conformation, where the small H substituent at C5 assumes a geared configuration with respect to the two relatively bulky methyl groups at C2′. The corresponding equilibrium S_1 structures (see parts c. and d. in Supplementary Figure 5) are computed by optimizing on the S_1 state a single equilibrium S_0 configuration obtained in the following way: (i) Selecting from the ASEC configuration (composed by 100 overlapping configurations) a single configuration component featuring the absorption maximum closest to the ASEC absorption maximum and (ii) performing a QM/MM geometry optimization in S_0 starting from this structure and relaxing both the QM part and the mobile solvent cavity (the MM cavity of Supplementary Figure 3b). The resulting optimized structure has then an absorption maximum very close to the ASEC absorption (these two values are indicated with green and red circles in Supplementary Figure 4 respectively).

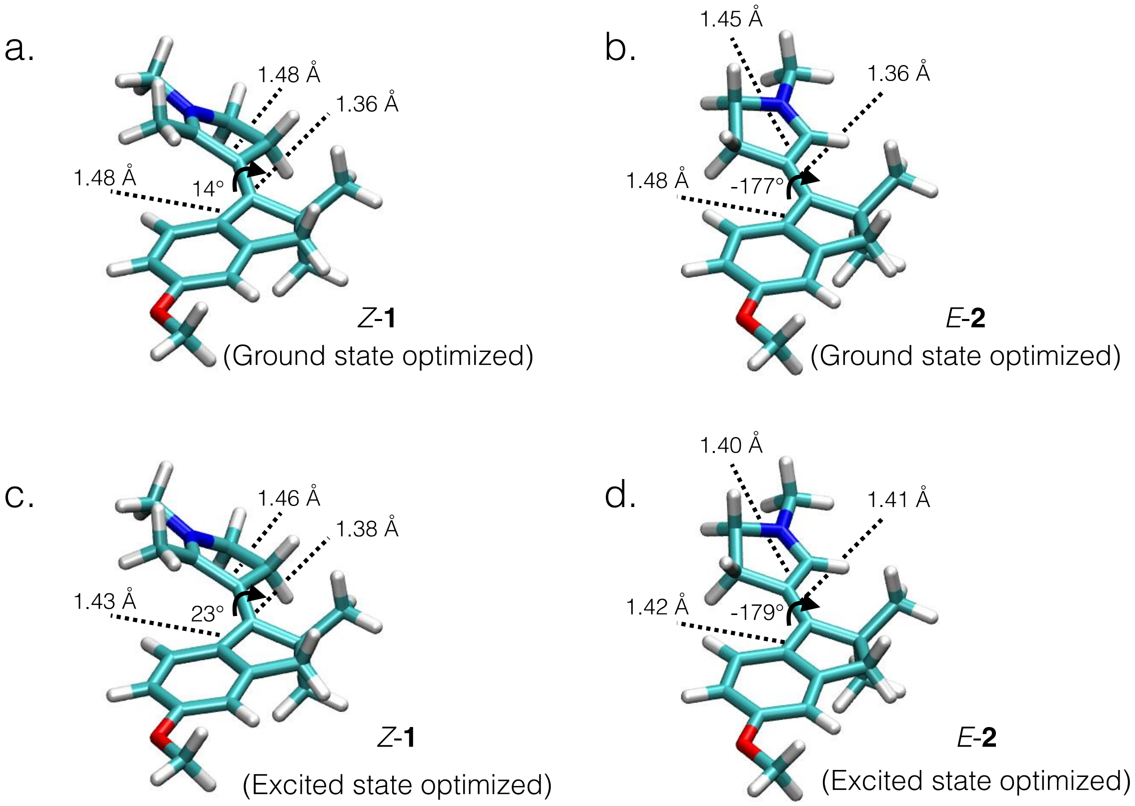

Supplementary Figure 5: Comparison of S_0 optimized and S_1 optimized geometries. Bond length values are in angstrom and dihedral angles are in degrees. The S_0 (ground state) structures are the geometrically optimized ASEC structures. The S_1 structures (excited state) are single configurations obtained by optimizing on S_1 state a specifically constructed single S_0 configuration. The corresponding Cartesian coordinates are given in the Supplementary Tables 1, 2, 4 and 5. Notice that the excited state structure of *E*-**2** has actually been optimized on the S_2 state as this state is actually the spectroscopic state at the CASSCF level (see Supplementary Figures 6c and 6d).

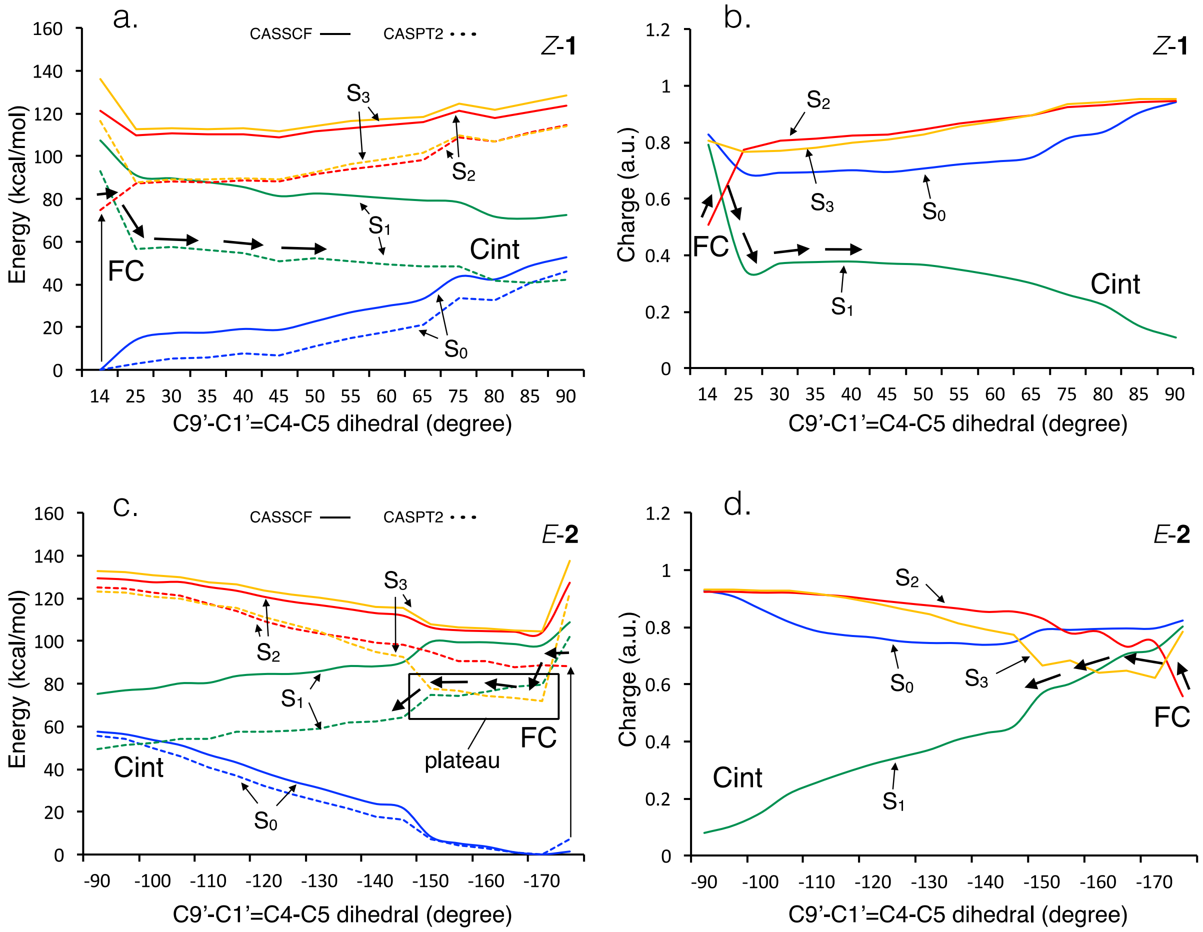

Supplementary Figure 6: Energy and charge profiles along the relaxed scan. The energy profiles of *Z*-**1** and *E*-**2** are shown in (a) and (c) respectively. Notice the flat or slightly uphill *E*-**2** reaction path region between -150 and -170 degrees (part c) which is absent in *Z*-**1**. The corresponding charge profiles are shown in (b) and (d). The charges are computed for the pyrroline fragment (see Figure 4 in the main text). S_0 to S_3 states are represented by blue, green, red and yellow colors respectively. CASSCF and CASPT2 energy profiles are shown in solid and dashed lines.

Supplementary Note 3: XMCQDPT2 energy profiles of *Z*-1 and *E*-2 trajectories.

Although the CASPT2 method accounts for dynamic electron correlation and results in better energies with respect to CASSCF, it fails to describe near degeneracy regions [17-19]. Due to this reason, the energy profiles along the trajectories were also computed at the extended multiconfigurational quasi-degenerate second order perturbation theory (XMCQDPT2) [19]. This was performed using the Firefly computer package [20]. The resulting XMCQDPT2 energy profiles are reported in the Supplementary Figure 7. By comparing these with the corresponding CASPT2 energies reported in Figure 4, one can clearly see that crossings between potential energy surfaces at CASPT2 level have now become avoided crossings.

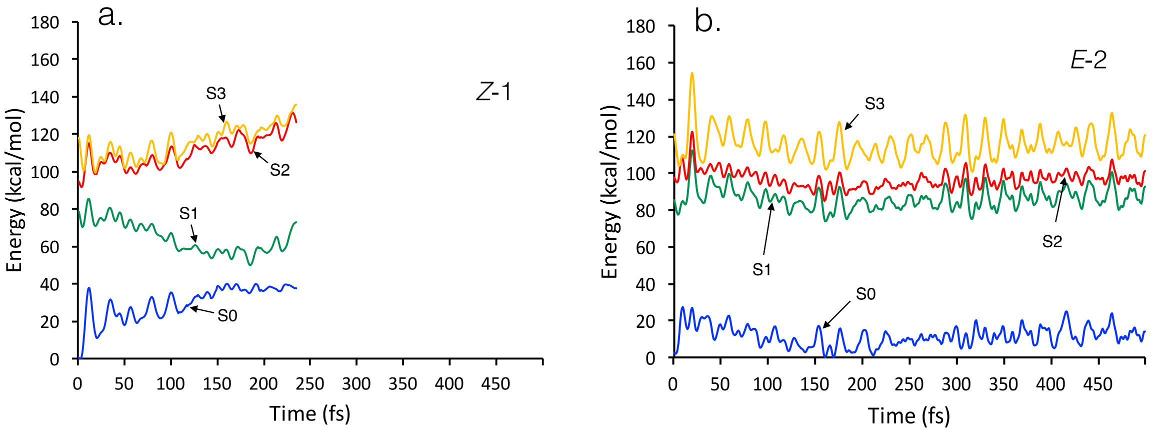

Supplementary Figure 7: XMCQDPT2 energy profiles. Energies of *Z*-**1** and *E*-**2** trajectories recomputed at XMCQDPT2 level of theory are shown in (a) and (b) respectively. Note that the S_1/S_0 crossing of *Z*-**1** at CASPT2 level is an avoided crossing at the XMCQDPT2 level.

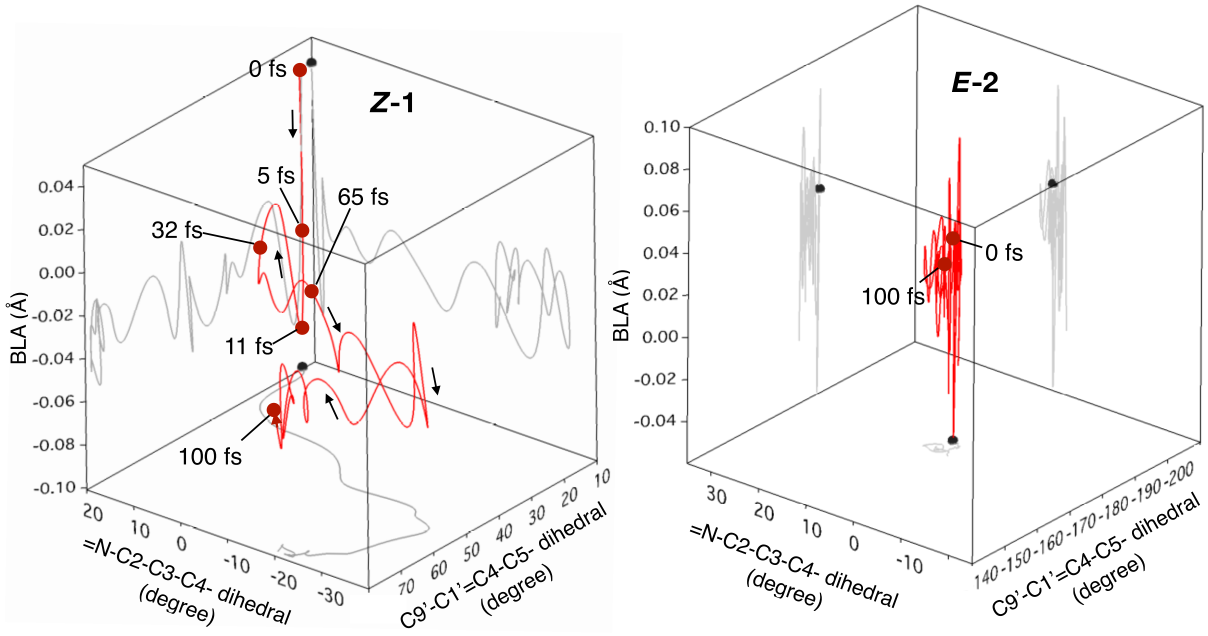

Supplementary Figure 8: Excited state geometrical progression along the isomerization coordinate. Left. First 100 fs evolution of the bond length alternation stretching coordinate (BLA), double bond isomerization coordinate (-C9'-C1'=C4-C5-) and pyrroline out-of-plane deformation coordinate (=N-C2-C3-C4-) along the FC trajectory of *Z*-**1**. Right. Same data for the FC trajectory of *E*-**2**. The times given along the *Z*-**1** coordinate show that the BLA mode is populated immediately, that the -C9'-C1'=C4-C5- isomerization activate only after 11 fs and that the out of plane deformation becomes active only after 32 fs. In contrast, along the *E*-**2** deformation coordinate, only BLA is active during the first 100 fs.

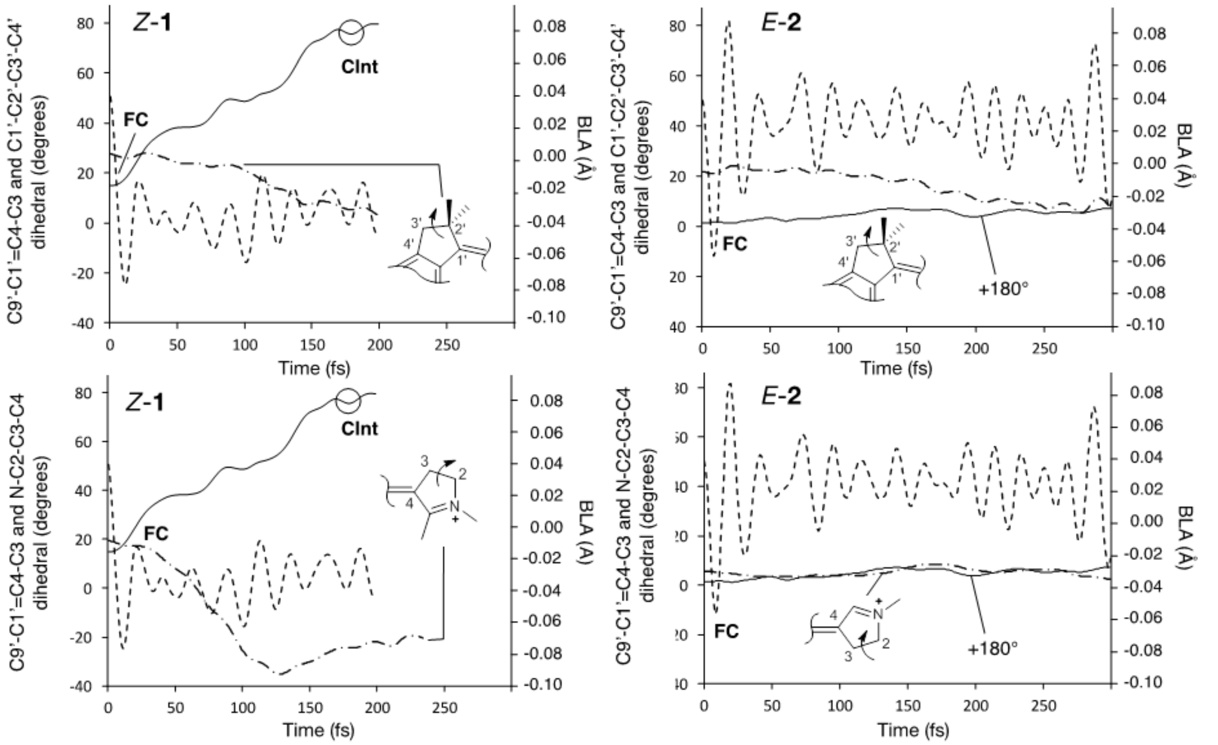

Supplementary Figure 9: Contribution of the excited state out-of-plane ring deformations along the isomerization coordinate of *Z*-1 and *E*-2. Upper panels. Progression along the reaction coordinate of *Z*-**1** (left) and *E*-**2** (right) of the out-of-plane deformation of the indanylidene five-membered ring (described by the C1'-C2'-C3'-C4' dihedral. See dashed-dotted line). The BLA stretching coordinate (dashed line) and -C9'-C1'=C4-C5- isomerization coordinates (full line) are reported for comparison. Lower panel. The same diagram for the out-of-plane deformation of the pyrroline ring (reflected by the =N-C2-C3-C4- dihedral, dashed-dotted line). Note that the coordinate evolution represented in the left and right panels for the pyrroline ring are associated to the left and right diagrams of Supplementary Figure 8, respectively.

Supplementary Table 1: Cartesian coordinates of the *Z*-**1** S_0 optimized structure

C	29.427736	29.015047	29.453060
C	28.353094	29.808386	29.055771
C	27.042068	29.326540	29.069389
H	26.234720	29.959850	28.754173
C	26.811008	28.030775	29.532990
C	27.881346	27.248123	30.008062
H	27.663351	26.274994	30.409748
C	29.180080	27.734888	29.979145
O	25.594677	27.457245	29.612226
C	24.462658	28.150959	29.148081
H	23.623228	27.489092	29.294422
H	24.545912	28.386135	28.093115
H	24.294603	29.063438	29.705294
H	29.971798	27.139429	30.394741
C	28.828794	31.192608	28.702818
C	30.676279	29.809011	29.394695
C	30.230665	31.290359	29.371790
H	28.165642	31.974518	29.055922
H	28.917070	31.306139	27.626455
C	30.070246	31.808727	30.812279
H	29.402081	31.177223	31.388664
H	29.655182	32.810318	30.797726
H	31.025177	31.843634	31.328596
C	31.117172	32.246498	28.564541
H	31.349665	31.839611	27.585114
H	32.045704	32.487732	29.064962
H	30.582869	33.178921	28.421772
C	31.961069	29.372066	29.375572
C	33.206722	30.202024	29.653011
C	34.253910	29.152920	30.019430
H	33.069016	30.905251	30.458687
H	33.533871	30.752256	28.783375
N	33.691620	27.907988	29.466423
H	34.382565	29.022623	31.085720
H	35.210709	29.332905	29.553942
C	32.454716	28.005838	29.111095
C	31.769289	26.896940	28.366224
C	34.560249	26.746569	29.319569
H	35.125771	26.630573	30.232975
H	35.227119	26.926857	28.488693
H	33.979187	25.856888	29.151308
H	31.595846	26.049477	29.017370
H	32.402798	26.588339	27.544870
H	30.832136	27.226871	27.952530

Supplementary Table 2: Cartesian coordinates of the *Z*-**1** S_1 optimized structure

C	29.478611	29.010237	29.456434
C	28.331758	29.839626	29.093540
C	27.003168	29.357302	29.062630
H	26.198664	29.994633	28.725220
C	26.795610	28.024231	29.549589
C	27.885401	27.225855	30.018177
H	27.649315	26.253811	30.394985
C	29.262287	27.685547	29.981424
O	25.580343	27.439127	29.614737
C	24.452908	28.146967	29.151068
H	23.610790	27.490547	29.297989
H	24.540546	28.387896	28.097271
H	24.299559	29.055479	29.718639
H	30.052952	27.095760	30.414497
C	28.816416	31.203014	28.707304
C	30.670883	29.809298	29.355606
C	30.224453	31.299061	29.354828
H	28.143267	31.998106	29.028223
H	28.906818	31.275629	27.624420
C	30.072731	31.808765	30.799540
H	29.400787	31.182549	31.379021
H	29.665161	32.813427	30.801729
H	31.028364	31.831104	31.314817
C	31.110331	32.252377	28.553373
H	31.349722	31.843212	27.577080
H	32.033680	32.475479	29.070047
H	30.591070	33.190750	28.408443
C	31.975153	29.345474	29.408428
C	33.196445	30.190834	29.671310
C	34.267429	29.169466	30.025785
H	33.064896	30.898703	30.468204
H	33.494579	30.719997	28.783045
N	33.724052	27.910759	29.449350
H	34.395496	29.027754	31.091988
H	35.222050	29.364971	29.560966
C	32.476509	28.004354	29.108549
C	31.779617	26.896610	28.358423
C	34.577068	26.727592	29.319557
H	35.135368	26.617800	30.240334
H	35.250496	26.887083	28.489338
H	33.964264	25.856531	29.161015
H	31.591927	26.056149	29.012939
H	32.415997	26.587065	27.542170
H	30.841890	27.225603	27.939508

Supplementary Table 3: Cartesian coordinates of the *Z*-**1** structure corresponding to S_1/S_0 CInt

C	29.360643	29.071505	29.568848
C	28.260340	29.866354	29.164026
C	26.981825	29.336222	29.113556
H	26.152252	29.936354	28.794754
C	26.806681	27.995792	29.492006
C	27.906853	27.189363	29.909672
H	27.701548	26.175528	30.198660
C	29.177883	27.704315	29.929436
O	25.637840	27.377434	29.488365
C	24.448166	28.064445	29.136616
H	23.652859	27.346380	29.244728
H	24.486977	28.407721	28.111046
H	24.274960	28.894585	29.806160
H	30.016998	27.106964	30.230576
C	28.733122	31.241146	28.776670
C	30.548122	29.805794	29.475569
C	30.197884	31.282515	29.276215
H	28.123070	32.031681	29.197611
H	28.694386	31.348358	27.696459
C	30.229271	31.924891	30.687883
H	29.556103	31.415417	31.370350
H	29.906160	32.954811	30.601866
H	31.225279	31.912127	31.109467
C	31.144526	32.045048	28.346001
H	31.163919	31.615486	27.350378
H	32.156292	32.050114	28.732159
H	30.809673	33.073402	28.261995
C	31.861877	29.260553	29.648380
C	32.650940	29.310095	30.956829
C	33.939465	28.540174	30.601316
H	32.127750	28.847309	31.784765
H	32.879391	30.330120	31.242371
N	33.814041	28.236063	29.187447
H	34.034286	27.620195	31.168482
H	34.822792	29.140419	30.784639
C	32.635105	28.657263	28.698578
C	32.255512	28.419687	27.266878
C	34.825729	27.455189	28.521114
H	35.123524	26.627489	29.156994
H	35.698132	28.058310	28.296904
H	34.453144	27.052402	27.593447
H	32.327614	27.373137	26.999374
H	32.900925	28.977314	26.603523
H	31.238836	28.729944	27.088476

Supplementary Table 4: Cartesian coordinates of the *E*-**2** S_0 optimized structure

C	29.092119	28.776308	29.366653
C	28.163679	29.781540	29.100937
C	26.790937	29.531029	29.063534
H	26.105968	30.325764	28.841839
C	26.344633	28.234262	29.313208
C	27.268768	27.215393	29.601870
H	26.898428	26.232040	29.828613
C	28.632425	27.474318	29.632898
O	25.040930	27.868948	29.327244
C	24.051413	28.863302	29.199936
H	23.102903	28.360374	29.318062
H	24.090462	29.341745	28.228796
H	24.147755	29.616806	29.971388
H	29.297756	26.678350	29.898491
C	28.858213	31.089035	28.844746
C	30.456417	29.345049	29.345020
C	30.306507	30.884087	29.363773
H	28.372530	31.927977	29.329241
H	28.875013	31.296627	27.779190
C	30.417307	31.377714	30.819372
H	29.652388	30.935833	31.449646
H	30.300447	32.455051	30.862261
H	31.387917	31.138300	31.243160
C	31.271341	31.675579	28.466698
H	31.409885	31.194220	27.504765
H	32.241478	31.835308	28.920485
H	30.857494	32.660725	28.288954
C	31.605434	28.630371	29.309742
C	31.781355	27.120552	29.217995
C	33.294712	26.919603	29.042772
H	31.234423	26.705078	28.382690
H	31.444231	26.634989	30.121305
N	33.849777	28.277933	29.188715
H	33.575043	26.556358	28.064548
H	33.744156	26.290388	29.795778
C	32.947765	29.178724	29.313869
H	33.234772	30.200325	29.425524
C	35.289471	28.516817	29.163610
H	35.696514	28.090034	28.260788
H	35.728622	28.028883	30.018923
H	35.477486	29.576378	29.190197

Supplementary Table 5: Cartesian coordinates of the *E*-**2** S_1 optimized structure

C	29.127041	28.786816	29.366087
C	28.145046	29.798162	29.098885
C	26.799252	29.542477	29.061085
H	26.105036	30.327629	28.839327
C	26.333200	28.229675	29.313650
C	27.281324	27.207024	29.603237
H	26.901791	26.230059	29.831375
C	28.623314	27.459341	29.635118
O	25.048584	27.870085	29.326664
C	24.050177	28.864208	29.198425
H	23.106674	28.355011	29.314800
H	24.091803	29.333876	28.224894
H	24.150864	29.611795	29.973295
H	29.299063	26.677317	29.899590
C	28.857885	31.090481	28.845022
C	30.410776	29.349861	29.346102
C	30.304121	30.883675	29.361713
H	28.366072	31.927181	29.324678
H	28.872540	31.289499	27.777081
C	30.417572	31.377602	30.819382
H	29.652498	30.936295	31.451849
H	30.300892	32.455738	30.858465
H	31.386551	31.132917	31.240705
C	31.269983	31.675877	28.465918
H	31.413830	31.195572	27.505217
H	32.237209	31.827381	28.920322
H	30.848768	32.658733	28.290668
C	31.647144	28.626986	29.310858
C	31.784555	27.122789	29.216576
C	33.301666	26.924782	29.042711
H	31.233976	26.702307	28.380728
H	31.438354	26.637695	30.122910
N	33.868053	28.258154	29.186489
H	33.559509	26.531237	28.065708
H	33.727596	26.267910	29.789068
C	32.914600	29.187862	29.318930
H	33.215759	30.203079	29.424820
C	35.286926	28.513054	29.164486
H	35.718063	28.098902	28.261795
H	35.756889	28.042315	30.020205
H	35.475655	29.571651	29.192142

Supplementary Table 6: Cartesian coordinates of the *E*-**2** structure corresponding to S_1/S_0 CInt

C	29.245024	28.911858	29.436100
C	28.286741	29.936071	29.254509
C	26.929726	29.654022	29.241571
H	26.210674	30.431774	29.083185
C	26.534451	28.323393	29.426418
C	27.489517	27.283352	29.605009
H	27.125116	26.285440	29.760410
C	28.834410	27.558313	29.610575
O	25.263527	27.923397	29.441935
C	24.212528	28.870495	29.342916
H	23.299259	28.306293	29.447578
H	24.223583	29.365423	28.382097
H	24.274347	29.603779	30.136019
H	29.558872	26.781904	29.759024
C	28.974545	31.253769	29.041811
C	30.538425	29.441856	29.354445
C	30.463535	30.962029	29.345037
H	28.559616	32.033859	29.668034
H	28.850140	31.564781	28.010656
C	30.829191	31.428919	30.776054
H	30.134907	31.038309	31.513947
H	30.789236	32.511284	30.810751
H	31.832893	31.127722	31.044621
C	31.412954	31.609272	28.332809
H	31.215942	31.246241	27.330458
H	32.446354	31.404774	28.582186
H	31.270227	32.683479	28.341173
C	31.744736	28.664049	29.298487
C	32.262825	28.023863	28.005194
C	33.498291	27.227083	28.489418
H	32.545345	28.762178	27.263594
H	31.517990	27.377012	27.556131
N	33.645546	27.595528	29.886858
H	34.396664	27.471144	27.938154
H	33.338757	26.157463	28.406147
C	32.623188	28.350281	30.285671
H	32.581646	28.671232	31.302780
C	34.711342	27.102928	30.716980
H	35.664227	27.530350	30.428740
H	34.769054	26.024750	30.643179
H	34.504005	27.352776	31.743305

Supplementary References

1. Paolino, M. *et al.* Design, Synthesis, and Dynamics of a Green Fluorescent Protein Fluorophore Mimic with an Ultrafast Switching Function. *J. Am. Chem. Soc.* **138,** 9807–9825 (2016).
2. Orozco-Gonzalez, Y. *et al.* An Average Solvent Electrostatic Configuration Protocol for QM/MM Free Energy Optimization: Implementation and Application to Rhodopsin Systems. *J. Chem. Theory Comput.* **13**, 6391-6404 (**2017**).
3. Georg, H. C. & Canuto, S. Electronic Properties of Water in Liquid Environment. A Sequential QM/MM Study Using the Free Energy Gradient Method. *J. Phys. Chem. B* **116,** 11247–11254 (2012).
4. Bistafa, C., Georg, H. C. & Canuto, S. Combining ab initio multiconfigurational and Free Energy Gradient methods to study the π–π* excited state structure and properties of uracil in water. *Comput. Theor. Chem.* **1040–1041,** 312–320 (2014).
5. Coutinho, K., Georg, H. C., Fonseca, T. L., Ludwig, V. & Canuto, S. An efficient statistically converged average configuration for solvent effects. *Chem. Phys. Lett.* **437,** 148–152 (2007).
6. Coutinho, K.; Rivelino, R.; Georg, H. C.; Canuto, S. In Solvation effects on molecules and biomolecules: Computational methods and applications; Canuto, S., Ed.; Springer: London, Vol. **6**, pp 159-189 (2008).
7. Okuyama-Yoshida, N., Nagaoka, M. & Yamabe, T. Transition-state optimization on free energy surface: Toward solution chemical reaction ergodography. *Int. J. Quantum Chem.* **70,** 95–103 (1998).
8. Okuyama-Yoshida, N., Kataoka, K., Nagaoka, M. & Yamabe, T. Structure optimization via free energy gradient method: Application to glycine zwitterion in aqueous solution. *J. Chem. Phys.* **113,** 3519–3524 (2000).
9. Hirao, H., Nagae, Y. & Nagaoka, M. Transition-state optimization by the free energy gradient method: Application to aqueous-phase Menshutkin reaction between ammonia and methyl chloride. *Chem. Phys. Lett.* **348,** 350–356 (2001).
10. Pronk, S. *et al.* GROMACS 4.5: a high-throughput and highly parallel open source molecular simulation toolkit. *Bioinformatics* **29,** 845–54 (2013).
11. Melaccio, F., Olivucci, M., Lindh, R. & Ferré, N. Unique QM/MM potential energy surface exploration using microiterations. *Int. J. Quantum Chem.* **111,** 3339–3346 (2011).
12. Aquilante, F. *et al.* MOLCAS 7 : The Next Generation. *J. Comput. Chem.* **31**, 224-47 (2010).
13. Roos, B. O. in *Advances in Chemical Physics, v. 69: ab initio Methods in Quantum Chemistry, part II* 399–445 (John Wiley & Sons, Inc., Hoboken, NJ, USA, 1987).
14. Jorgensen, W. L., Maxwell, D. S. & Tirado-Rives, J. Development and testing of the OPLS all-atom force field on conformational energetics and properties of organic liquids. *J. Am. Chem. Soc.* **118,** 11225–11236 (1996).
15. Andersson, K., Malmqvist, P.-Å. & Roos, B. O. Second-order perturbation theory with a complete active space self-consistent field reference function. *J. Chem. Phys.* **96,** 1218–1226 (1992).
16. Schapiro, I. *et al.* The ultrafast photoisomerizations of rhodopsin and bathorhodopsin are modulated by bond length alternation and HOOP driven electronic effects. *J. Am. Chem. Soc.* **133,** 3354–3364 (2011).
17. Gozem, S. *et al.* Dynamic electron correlation effects on the ground state potential energy surface of a retinal chromophore model. *J. Chem. Theory Comput.* **8,** 4069–4080 (2012).
18. Malrieu, J.-P., Heully, J.-L. & Zaitsevskii, A. Multiconfigurational second-order perturbative methods: Overview and comparison of basic properties. *Theor. Chim. Acta* **90,** 167–187 (1995).
19. Granovsky, A. A., Extended multi-configuration quasi-degenerate perturbation theory: The new approach to multi-state multi-reference perturbation theory. *J. Chem. Phys.*, **134**, 214113 (2011).
20. Granovsky, A. A., Firefly, version 8.0.0. http://classic.chem.msu.su/gran/firefly/index.html. Available at: http://classic.chem.msu.su/gran/firefly/index.html [Accessed 2015].
